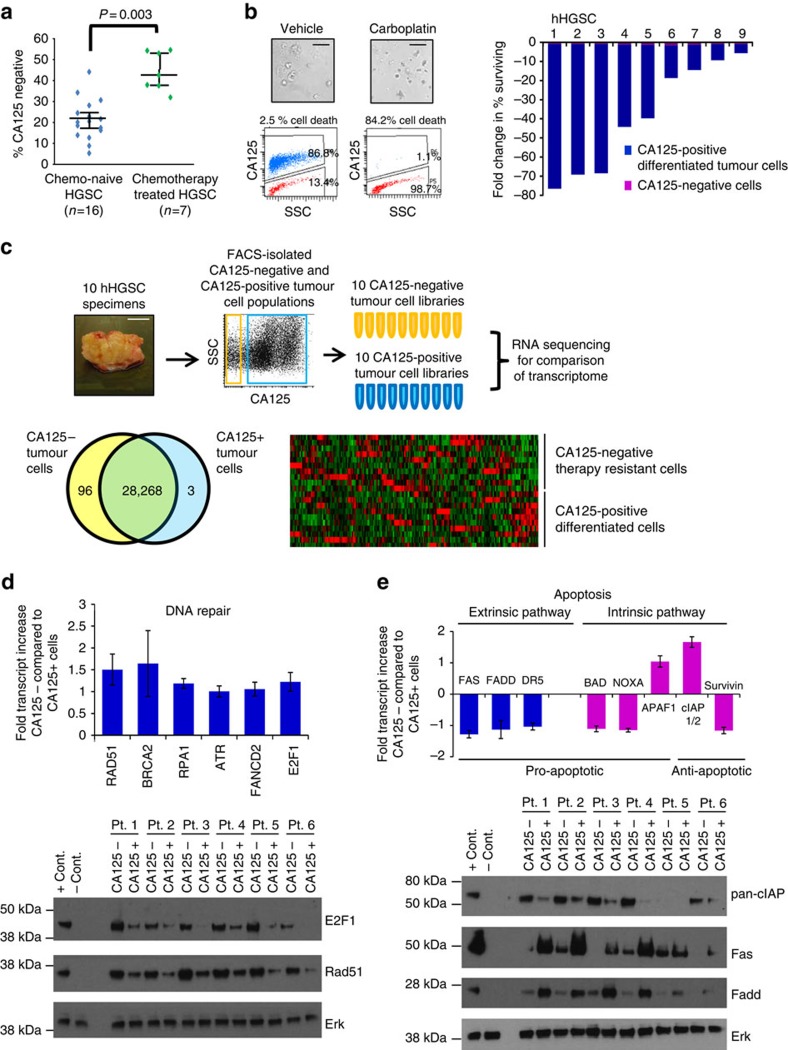# Corrigendum: An apoptosis-enhancing drug overcomes platinum resistance in a tumour-initiating subpopulation of ovarian cancer

**DOI:** 10.1038/ncomms10703

**Published:** 2016-02-05

**Authors:** D. M. Janzen, E. Tiourin, J. A. Salehi, D. Y. Paik, J. Lu, M. Pellegrini, S. Memarzadeh

Nature Communications 6, Article number: 795610.1038/ncomms8956 (2015); Published 08032015; Updated 02052016

After reanalysing the RNA sequencing data presented in Supplementary Data 3 of this Article, we have discovered that 341,056 of the 730,620 expression values were inadvertently paired with incorrect gene names. These incorrect values were used in Figure 3c–e and Supplementary Tables 4,5,7 and 8 and led us to conclude that there are differences in the expression of DNA repair and apoptosis genes between CA125-negative and CA125-positive cells. We have now reanalysed the corrected data, and present a revised version of Figure 3 below. We find that, of the DNA repair and apoptosis genes reported in Figure 3, none display a statistically significant difference in expression between CA125-negative and CA125-positive cells at the transcript level. However, at the protein level, cIAPs, the target proteins for Birinapant, are nevertheless expressed at substantially higher levels by CA125-negative cells, implying that the regulatory mechanisms of cIAP protein expression may be different from transcriptional regulation in CA125-negative cells. Therefore, the overall conclusion in regard to therapy, that birinapant strongly sensitizes CA125-negative cells to carboplatin-induced death due to their high cIAP protein expression, remains unaffected by the error in RNA-sequencing data analysis. Corrected versions of Supplementary Data 3 and Supplementary Tables 4,5,7 and 8 are available from the HTML version of this Corrigendum.

## Supplementary Material

Supplementary Information Supplementary Table 4

Supplementary Information Supplementary Table 5

Supplementary Information Supplementary Table 7

Supplementary Information Supplementary Table 8

Supplementary Data 3

## Figures and Tables

**Figure 3:**