# Supplementary material for: Corrigendum: An apoptosis-enhancing drug overcomes platinum resistance in a tumour-initiating subpopulation of ovarian cancer
Source: Nat Commun. 2016 Feb 5;7:10703. doi: 10.1038/ncomms10703 (PMC4748230; doi:10.1038/ncomms10703)
Supplement: Supplementary file 1 [file ncomms10703-s1.docx]

**Supplementary Table 4. Fold change in drug efflux transcripts**

| **Gene** | **Fold up or Down in CA125 - compared to CA125 + population** | **Paired t test** |
| --- | --- | --- |
| ATP7A | 1.09 | 0.66 |
| ATP7B | 1.25 | 0.84 |
| SLC31A1 | 1.11 | 0.53 |
| GCSH | 1.08 | 0.47 |
| MGST3 | 1.08 | 0.50 |
| ABCB1 | -1.03 | 0.08 |
| ABCC1 | -1.07 | 0.19 |
| ABCC2 | 2.52 | 0.24 |
| ABCC3 | 1.01 | 0.04 |
| ABCC4 | 1.19 | 0.28 |
| ABCC5 | 1.29 | 0.78 |
| ABCC6 | -1.10 | 0.59 |
| VDAC1 | 1.02 | 0.66 |
| MTF1 | 1.13 | 0.39 |
| MT1H | -1.01 | 0.79 |
