# Supplementary material for: Corrigendum: An apoptosis-enhancing drug overcomes platinum resistance in a tumour-initiating subpopulation of ovarian cancer
Source: Nat Commun. 2016 Feb 5;7:10703. doi: 10.1038/ncomms10703 (PMC4748230; doi:10.1038/ncomms10703)
Supplement: Supplementary file 2 [file ncomms10703-s2.docx]

**Supplementary Table 5. Fold change in autophagy transcripts**

| **Gene** | **Fold up or Down in CA125 - compared to CA125 + population** | **Paired t test** |
| --- | --- | --- |
| IFNG | 1.68 | 0.29 |
| ULK1 | 1.28 | 0.98 |
| ATG13 | 1.07 | 0.91 |
| BECN1 | 1.04 | 0.85 |
| ATG12 | -1.00 | 0.81 |
| ATG14 | 1.00 | 0.55 |
| ATG7 | 1.14 | 0.40 |
| ATG10 | -1.02 | 0.87 |
| ATG4A | 1.02 | 0.47 |
