# Supplementary material for: Corrigendum: An apoptosis-enhancing drug overcomes platinum resistance in a tumour-initiating subpopulation of ovarian cancer
Source: Nat Commun. 2016 Feb 5;7:10703. doi: 10.1038/ncomms10703 (PMC4748230; doi:10.1038/ncomms10703)
Supplement: Supplementary file 3 [file ncomms10703-s3.docx]

**Supplementary Table 7. Secreted proteins transcripts highly expressed in CA125- cells**

| ID | Gene Symbol | Gene Description | Fold increase over average |
| --- | --- | --- | --- |
| ENSG00000166710 | B2M | beta-2-microglobulin | 184.2138 |
| ENSG00000120885 | CLU | clusterin | 121.3041 |
| ENSG00000169429 | IL8 | interleukin 8 | 80.80418 |
| ENSG00000182718 | ANXA2 | annexin A2 | 74.79547 |
| ENSG00000124107 | SLPI | secretory leukocyte peptidase inhibitor | 57.0789 |
| ENSG00000135046 | ANXA1 | annexin A1 | 55.26921 |
| ENSG00000163739 | CXCL1 | chemokine (C-X-C motif) ligand 1 | 35.29779 |
| ENSG00000058085 | LAMC2 | laminin, gamma 2 | 32.03763 |
| ENSG00000108679 | LGALS3BP | lectin, galactoside-binding, soluble, 3 binding protein | 26.7803 |
| ENSG00000113140 | SPARC | secreted protein, acidic, cysteine-rich (osteonectin) | 25.87084 |
| ENSG00000149131 | SERPING1 | serpin peptidase inhibitor, clade G, member 1 | 25.44536 |
| ENSG00000101443 | WFDC2 | WAP four-disulfide core domain 2 | 23.56747 |
| ENSG00000118785 | SPP1 | secreted phosphoprotein 1 | 20.85439 |
| ENSG00000185499 | MUC1 | mucin 1, cell surface associated | 19.40105 |
| ENSG00000102265 | TIMP1 | TIMP metallopeptidase inhibitor 1 | 18.98433 |
| ENSG00000137673 | MMP7 | matrix metallopeptidase 7 (matrilysin, uterine) | 17.19641 |
| ENSG00000169245 | CXCL10 | chemokine (C-X-C motif) ligand 10 | 16.03441 |
| ENSG00000115414 | FN1 | fibronectin 1 | 15.6301 |
| ENSG00000125730 | C3 | complement component 3 | 13.81717 |
| ENSG00000167244 | IGF2 | insulin-like growth factor 2 (somatomedin A) | 13.5789 |
| ENSG00000167755 | KLK6 | kallikrein-related peptidase 6 | 12.21314 |
| ENSG00000257017 | HP | haptoglobin | 11.33771 |
| ENSG00000115009 | CCL20 | chemokine (C-C motif) ligand 20 | 10.96991 |
| ENSG00000175899 | A2M | alpha-2-macroglobulin | 10.66031 |
| ENSG00000133048 | CHI3L1 | chitinase 3-like 1 (cartilage glycoprotein-39) | 10.59176 |
| ENSG00000169035 | KLK7 | kallikrein-related peptidase 7 | 10.54181 |
| ENSG00000205403 | CFI | complement factor I | 10.39814 |
| ENSG00000008517 | IL32 | interleukin 32 | 9.921979 |
| ENSG00000136689 | IL1RN | interleukin 1 receptor antagonist | 9.283687 |
| ENSG00000100097 | LGALS1 | lectin, galactoside-binding, soluble, 1 | 9.134792 |
| ENSG00000164692 | COL1A2 | collagen, type I, alpha 2 | 8.986852 |
| ENSG00000053747 | LAMA3 | laminin, alpha 3 | 8.8568 |
| ENSG00000110492 | MDK | midkine (neurite growth-promoting factor 2) | 8.465323 |
| ENSG00000078401 | EDN1 | endothelin 1 | 8.329528 |
| ENSG00000030582 | GRN | granulin | 8.180396 |
| ENSG00000104368 | PLAT | plasminogen activator, tissue | 8.082647 |
| ENSG00000148346 | LCN2 | lipocalin 2 | 7.493953 |
| ENSG00000129538 | RNASE1 | ribonuclease, RNase A family, 1 (pancreatic) | 7.477958 |
| ENSG00000047457 | CP | ceruloplasmin (ferroxidase) | 7.325464 |
| ENSG00000108821 | COL1A1 | collagen, type I, alpha 1 | 6.944076 |
| ENSG00000081041 | CXCL2 | chemokine (C-X-C motif) ligand 2 | 6.62555 |
| ENSG00000129277 | CCL4 | chemokine (C-C motif) ligand 4 | 6.485083 |
| ENSG00000021355 | SERPINB1 | serpin peptidase inhibitor, clade B, member 1 | 6.424642 |
| ENSG00000211445 | GPX3 | glutathione peroxidase 3 (plasma) | 6.423557 |
|  |  |  |  |
| **Supplementary Table 7. Continued** | | | |
| ENSG00000122861 | PLAU | plasminogen activator, urokinase | 5.702007 |
| ENSG00000168542 | COL3A1 | collagen, type III, alpha 1 | 5.624754 |
| ENSG00000115461 | IGFBP5 | insulin-like growth factor binding protein 5 | 5.616434 |
| ENSG00000163734 | CXCL3 | chemokine (C-X-C motif) ligand 3 | 5.430026 |
| ENSG00000196878 | LAMB3 | laminin, beta 3 | 5.27229 |
| ENSG00000232810 | TNF | tumor necrosis factor | 5.018159 |
| ENSG00000052344 | PRSS8 | protease, serine, 8 | 4.648191 |
| ENSG00000152268 | SPON1 | spondin 1, extracellular matrix protein | 4.620804 |
| ENSG00000100342 | APOL1 | apolipoprotein L, 1 | 4.567049 |
| ENSG00000197249 | SERPINA1 | serpin peptidase inhibitor, clade A , member 1 | 4.540708 |
| ENSG00000106366 | SERPINE1 | serpin peptidase inhibitor, clade E, member 1 | 4.487746 |
| ENSG00000135862 | LAMC1 | laminin, gamma 1 (formerly LAMB2) | 4.360297 |
| ENSG00000166670 | MMP10 | matrix metallopeptidase 10 | 4.150368 |
| ENSG00000243649 | CFB | complement factor B | 3.968198 |
| ENSG00000117122 | MFAP2 | microfibrillar-associated protein 2 | 3.914854 |
| ENSG00000163430 | FSTL1 | follistatin-like 1 | 3.802715 |
| ENSG00000011465 | DCN | decorin | 3.799718 |
| ENSG00000146674 | IGFBP3 | insulin-like growth factor binding protein 3 | 3.612927 |
| ENSG00000176945 | MUC20 | mucin 20, cell surface associated | 3.584843 |
| ENSG00000125538 | IL1B | interleukin 1, beta | 3.575904 |
| ENSG00000169218 | RSPO1 | R-spondin 1 | 3.355045 |
| ENSG00000139329 | LUM | lumican | 3.279579 |
| ENSG00000006075 | CCL3 | chemokine (C-C motif) ligand 3 | 3.266068 |
| ENSG00000129451 | KLK10 | kallikrein-related peptidase 10 | 3.247051 |
| ENSG00000168081 | PNOC | prepronociceptin | 3.044962 |
