# Supplementary material for: Corrigendum: An apoptosis-enhancing drug overcomes platinum resistance in a tumour-initiating subpopulation of ovarian cancer
Source: Nat Commun. 2016 Feb 5;7:10703. doi: 10.1038/ncomms10703 (PMC4748230; doi:10.1038/ncomms10703)
Supplement: Supplementary file 4 [file ncomms10703-s4.docx]

**Supplementary Table 8. Cell surface transcripts highly expressed in CA125- cells**

| ID | Gene Symbol | Gene Description | Fold increase over average |
| --- | --- | --- | --- |
| ENSG00000139644 | TMBIM6 | transmembrane BAX inhibitor motif containing 6 | 101.6694 |
| ENSG00000019582 | CD74 | CD74 molecule | 96.10164 |
| ENSG00000165949 | IFI27 | interferon, alpha-inducible protein 27 | 63.34793 |
| ENSG00000013588 | GPRC5A | G protein-coupled receptor, family C, group 5, member A | 61.71585 |
| ENSG00000169908 | TM4SF1 | transmembrane 4 L six family member 1 | 55.28075 |
| ENSG00000142089 | IFITM3 | interferon induced transmembrane protein 3 | 48.76032 |
| ENSG00000010278 | CD9 | CD9 molecule | 47.73234 |
| ENSG00000124145 | SDC4 | syndecan 4 | 36.32 |
| ENSG00000135404 | CD63 | CD63 molecule | 34.58436 |
| ENSG00000185885 | IFITM1 | interferon induced transmembrane protein 1 | 33.69365 |
| ENSG00000011422 | PLAUR | plasminogen activator, urokinase receptor | 31.3331 |
| ENSG00000110195 | FOLR1 | folate receptor 1 (adult) | 29.81204 |
| ENSG00000005022 | SLC25A5 | solute carrier family 25 , member 5 | 29.05286 |
| ENSG00000118705 | RPN2 | ribophorin II | 27.42461 |
| ENSG00000135535 | CD164 | CD164 molecule, sialomucin | 23.6551 |
| ENSG00000185825 | BCAP31 | B-cell receptor-associated protein 31 | 21.20962 |
| ENSG00000116209 | TMEM59 | transmembrane protein 59 | 21.09407 |
| ENSG00000169242 | EFNA1 | ephrin-A1 | 20.84975 |
| ENSG00000185499 | MUC1 | mucin 1, cell surface associated | 19.40105 |
| ENSG00000137575 | SDCBP | syndecan binding protein (syntenin) | 19.33312 |
| ENSG00000136235 | GPNMB | glycoprotein (transmembrane) nmb | 17.89397 |
| ENSG00000085063 | CD59 | CD59 molecule, complement regulatory protein | 17.58239 |
| ENSG00000170348 | TMED10 | transmembrane emp24-like trafficking protein 10 (yeast) | 17.3501 |
| ENSG00000105855 | ITGB8 | integrin, beta 8 | 16.45394 |
| ENSG00000185201 | IFITM2 | interferon induced transmembrane protein 2 | 16.39686 |
| ENSG00000189143 | CLDN4 | claudin 4 | 15.46918 |
| ENSG00000167642 | SPINT2 | serine peptidase inhibitor, Kunitz type, 2 | 15.46766 |
| ENSG00000125991 | ERGIC3 | ERGIC and golgi 3 | 15.32207 |
| ENSG00000134531 | EMP1 | epithelial membrane protein 1 | 14.88054 |
| ENSG00000039068 | CDH1 | cadherin 1, type 1, E-cadherin (epithelial) | 14.64162 |
| ENSG00000234745 | HLA-B | major histocompatibility complex, class I, B | 14.13077 |
| ENSG00000134910 | STT3A | STT3A | 14.04255 |
| ENSG00000197747 | S100A10 | S100 calcium binding protein A10 | 13.95846 |
| ENSG00000129562 | DAD1 | defender against cell death 1 | 13.9006 |
| ENSG00000181061 | HIGD1A | HIG1 hypoxia inducible domain family, member 1A | 13.83861 |
| ENSG00000130164 | LDLR | low density lipoprotein receptor | 13.66937 |
| ENSG00000137648 | TMPRSS4 | transmembrane protease, serine 4 | 13.25225 |
| ENSG00000117394 | SLC2A1 | solute carrier family 2, member 1 | 12.99304 |
| ENSG00000086598 | TMED2 | transmembrane emp24 domain trafficking protein 2 | 12.93232 |
| ENSG00000154639 | CXADR | coxsackie virus and adenovirus receptor | 12.63246 |
| ENSG00000143153 | ATP1B1 | ATPase, Na+/K+ transporting, beta 1 polypeptide | 11.3489 |
| ENSG00000125304 | TM9SF2 | transmembrane 9 superfamily member 2 | 11.11332 |
| ENSG00000213853 | EMP2 | epithelial membrane protein 2 | 10.77021 |
|  |  |  |  |
| **Supplementary Table 8. Cell surface transcripts highly expressed in CA125- cells** | | | |
|  | | | |
| ENSG00000090339 | ICAM1 | intercellular adhesion molecule 1 | 10.73906 |
| ENSG00000172270 | BSG | basigin | 10.61452 |
| ENSG00000112149 | CD83 | CD83 molecule | 10.41832 |
| ENSG00000113361 | CDH6 | cadherin 6, type 2, K-cadherin (fetal kidney) | 9.882616 |
| ENSG00000196776 | CD47 | CD47 molecule | 9.541996 |
| ENSG00000114638 | UPK1B | uroplakin 1B | 9.348166 |
| ENSG00000105426 | PTPRS | protein tyrosine phosphatase, receptor type, S | 9.341324 |
| ENSG00000204525 | HLA-C | major histocompatibility complex, class I, C | 9.212441 |
| ENSG00000206503 | HLA-A | major histocompatibility complex, class I, A | 9.111571 |
| ENSG00000134294 | SLC38A2 | solute carrier family 38, member 2 | 8.998898 |
| ENSG00000150093 | ITGB1 | integrin, beta 1 | 8.790971 |
| ENSG00000231389 | HLA-DPA1 | major histocompatibility complex, class II, DP alpha 1 | 8.737447 |
| ENSG00000116857 | TMEM9 | transmembrane protein 9 | 8.643996 |
| ENSG00000113946 | CLDN16 | claudin 16 | 8.628852 |
| ENSG00000111897 | SERINC1 | serine incorporator 1 | 8.541881 |
| ENSG00000171105 | INSR | insulin receptor | 8.439662 |
| ENSG00000111371 | SLC38A1 | solute carrier family 38, member 1 | 8.318505 |
| ENSG00000138449 | SLC40A1 | solute carrier family 40, member 1 | 8.255242 |
| ENSG00000179820 | MYADM | myeloid-associated differentiation marker | 8.224238 |
| ENSG00000196126 | HLA-DRB1 | major histocompatibility complex, class II, DR beta 1 | 8.130884 |
| ENSG00000184697 | CLDN6 | claudin 6 | 8.05851 |
| ENSG00000109133 | TMEM33 | transmembrane protein 33 | 7.928815 |
| ENSG00000135926 | TMBIM1 | transmembrane BAX inhibitor motif containing 1 | 7.868508 |
| ENSG00000143727 | ACP1 | acid phosphatase 1, soluble | 7.671318 |
| ENSG00000157765 | SLC34A2 | solute carrier family 34, member 2 | 7.60847 |
| ENSG00000117335 | CD46 | CD46 molecule, complement regulatory protein | 7.575767 |
| ENSG00000129353 | SLC44A2 | solute carrier family 44 (choline transporter), member 2 | 7.49459 |
| ENSG00000126709 | IFI6 | interferon, alpha-inducible protein 6 | 7.494433 |
| ENSG00000181885 | CLDN7 | claudin 7 | 7.108435 |
| ENSG00000117472 | TSPAN1 | tetraspanin 1 | 6.966931 |
| ENSG00000144136 | SLC20A1 | solute carrier family 20, member 1 | 6.854106 |
| ENSG00000008282 | SYPL1 | synaptophysin-like 1 | 6.683794 |
| ENSG00000072274 | TFRC | transferrin receptor | 6.577753 |
| ENSG00000129625 | REEP5 | receptor accessory protein 5 | 6.570635 |
| ENSG00000111843 | TMEM14C | transmembrane protein 14C | 6.511298 |
| ENSG00000143183 | TMCO1 | transmembrane and coiled-coil domains 1 | 6.49628 |
| ENSG00000091317 | CMTM6 | CKLF-like MARVEL transmembrane domain containing 6 | 6.295148 |
| ENSG00000167601 | AXL | AXL receptor tyrosine kinase | 6.167164 |
| ENSG00000147676 | MAL2 | mal, T-cell differentiation protein 2 (gene/pseudogene) | 6.135816 |
| ENSG00000026508 | CD44 | CD44 molecule (Indian blood group) | 6.135642 |
| ENSG00000170458 | CD14 | CD14 molecule | 6.130958 |
| ENSG00000184292 | TACSTD2 | tumor-associated calcium signal transducer 2 | 6.065425 |
| ENSG00000132824 | SERINC3 | serine incorporator 3 | 6.064649 |
| ENSG00000104341 | LAPTM4B | lysosomal protein transmembrane 4 beta | 6.039147 |
| ENSG00000162736 | NCSTN | nicastrin | 5.880781 |
| ENSG00000134825 | TMEM258 | transmembrane protein 258 | 5.862424 |
|  |  |  |  |
| **Supplementary Table 8. Cell surface transcripts highly expressed in CA125- cells** | | | |
| ENSG00000085117 | CD82 | CD82 molecule | 5.840293 |
| ENSG00000102158 | MAGT1 | magnesium transporter 1 | 5.832943 |
| ENSG00000137207 | YIPF3 | Yip1 domain family, member 3 | 5.753293 |
| ENSG00000163814 | CDCP1 | CUB domain containing protein 1 | 5.734674 |
| ENSG00000168542 | COL3A1 | collagen, type III, alpha 1 | 5.624754 |
| ENSG00000163347 | CLDN1 | claudin 1 | 5.472585 |
| ENSG00000162366 | PDZK1IP1 | PDZK1 interacting protein 1 | 5.457715 |
| ENSG00000110660 | SLC35F2 | solute carrier family 35, member F2 | 5.367756 |
| ENSG00000139163 | ETNK1 | ethanolamine kinase 1 | 5.294934 |
| ENSG00000168003 | SLC3A2 | solute carrier family 3 , member 2 | 5.196647 |
| ENSG00000126353 | CCR7 | chemokine (C-C motif) receptor 7 | 5.157968 |
| ENSG00000143570 | SLC39A1 | solute carrier family 39 (zinc transporter), member 1 | 5.127779 |
| ENSG00000075420 | FNDC3B | fibronectin type III domain containing 3B | 5.116679 |
| ENSG00000005486 | RHBDD2 | rhomboid domain containing 2 | 5.098798 |
| ENSG00000163399 | ATP1A1 | ATPase, Na+/K+ transporting, alpha 1 polypeptide | 5.092461 |
| ENSG00000000003 | TSPAN6 | tetraspanin 6 | 5.052314 |
| ENSG00000074696 | PTPLAD1 | protein tyrosine phosphatase-like A domain containing 1 | 5.012757 |
| ENSG00000027697 | IFNGR1 | interferon gamma receptor 1 | 4.979089 |
| ENSG00000089063 | TMEM230 | transmembrane protein 230 | 4.794139 |
| ENSG00000158769 | F11R | F11 receptor | 4.731782 |
| ENSG00000080822 | CLDND1 | claudin domain containing 1 | 4.71654 |
| ENSG00000137210 | TMEM14B | transmembrane protein 14B | 4.711594 |
| ENSG00000223865 | HLA-DPB1 | major histocompatibility complex, class II, DP beta 1 | 4.632609 |
| ENSG00000100342 | APOL1 | apolipoprotein L, 1 | 4.567049 |
| ENSG00000185475 | TMEM179B | transmembrane protein 179B | 4.512045 |
| ENSG00000162896 | PIGR | polymeric immunoglobulin receptor | 4.508271 |
| ENSG00000134291 | TMEM106C | transmembrane protein 106C | 4.498658 |
| ENSG00000012660 | ELOVL5 | ELOVL fatty acid elongase 5 | 4.469504 |
| ENSG00000101558 | VAPA | VAMP associated protein A | 4.45556 |
| ENSG00000168615 | ADAM9 | ADAM metallopeptidase domain 9 | 4.452483 |
| ENSG00000142949 | PTPRF | protein tyrosine phosphatase, receptor type, F | 4.435171 |
| ENSG00000058668 | ATP2B4 | ATPase, Ca++ transporting, plasma membrane 4 | 4.41905 |
| ENSG00000204385 | SLC44A4 | solute carrier family 44, member 4 | 4.416825 |
| ENSG00000165029 | ABCA1 | ATP-binding cassette, sub-family A (ABC1), member 1 | 4.399048 |
| ENSG00000137642 | SORL1 | sortilin-related receptor, L(DLR class) | 4.379822 |
| ENSG00000170876 | TMEM43 | transmembrane protein 43 | 4.333676 |
| ENSG00000108639 | SYNGR2 | synaptogyrin 2 | 4.277964 |
| ENSG00000003056 | M6PR | mannose-6-phosphate receptor (cation dependent) | 4.233111 |
| ENSG00000112378 | PERP | PERP, TP53 apoptosis effector | 4.216953 |
| ENSG00000162695 | SLC30A7 | solute carrier family 30 (zinc transporter), member 7 | 4.212633 |
| ENSG00000080815 | PSEN1 | presenilin 1 | 4.14614 |
| ENSG00000134247 | PTGFRN | prostaglandin F2 receptor inhibitor | 4.129098 |
| ENSG00000106565 | TMEM176B | transmembrane protein 176B | 4.0718 |
| ENSG00000140497 | SCAMP2 | secretory carrier membrane protein 2 | 4.063287 |
| ENSG00000197822 | OCLN | occludin | 4.035511 |
| **Supplementary Table 8. Cell surface transcripts highly expressed in CA125- cells** | | | |
| ENSG00000136156 | ITM2B | integral membrane protein 2B | 3.987404 |
| ENSG00000105974 | CAV1 | caveolin 1, caveolae protein, 22kDa | 3.986552 |
| ENSG00000143771 | CNIH4 | cornichon family AMPA receptor auxiliary protein 4 | 3.927053 |
| ENSG00000100979 | PLTP | phospholipid transfer protein | 3.897151 |
| ENSG00000059804 | SLC2A3 | solute carrier family 2, member 3 | 3.877508 |
| ENSG00000132965 | ALOX5AP | arachidonate 5-lipoxygenase-activating protein | 3.862201 |
| ENSG00000168701 | TMEM208 | transmembrane protein 208 | 3.859491 |
| ENSG00000142166 | IFNAR1 | interferon (alpha, beta and omega) receptor 1 | 3.845693 |
| ENSG00000205155 | PSENEN | presenilin enhancer gamma secretase subunit | 3.830504 |
| ENSG00000034677 | RNF19A | ring finger protein 19A, RBR E3 ubiquitin protein ligase | 3.821623 |
| ENSG00000133195 | SLC39A11 | solute carrier family 39, member 11 | 3.797819 |
| ENSG00000100647 | KIAA0247 | KIAA0247 | 3.785539 |
| ENSG00000006327 | TNFRSF12A | tumor necrosis factor receptor superfamily, member 12A | 3.78171 |
| ENSG00000133872 | TMEM66 | transmembrane protein 66 | 3.6959 |
| ENSG00000137845 | ADAM10 | ADAM metallopeptidase domain 10 | 3.673015 |
| ENSG00000128335 | APOL2 | apolipoprotein L, 2 | 3.662301 |
| ENSG00000108219 | TSPAN14 | tetraspanin 14 | 3.630095 |
| ENSG00000101294 | HM13 | histocompatibility (minor) 13 | 3.62827 |
| ENSG00000116521 | SCAMP3 | secretory carrier membrane protein 3 | 3.626574 |
| ENSG00000114023 | FAM162A | family with sequence similarity 162, member A | 3.601581 |
| ENSG00000146066 | HIGD2A | HIG1 hypoxia inducible domain family, member 2A | 3.586989 |
| ENSG00000176945 | MUC20 | mucin 20, cell surface associated | 3.584843 |
| ENSG00000184743 | ATL3 | atlastin GTPase 3 | 3.51435 |
| ENSG00000119888 | EPCAM | epithelial cell adhesion molecule | 3.50729 |
| ENSG00000169508 | GPR183 | G protein-coupled receptor 183 | 3.503245 |
| ENSG00000103978 | TMEM87A | transmembrane protein 87A | 3.49856 |
| ENSG00000204257 | HLA-DMA | major histocompatibility complex, class II, DM alpha | 3.489599 |
| ENSG00000142627 | EPHA2 | EPH receptor A2 | 3.483071 |
| ENSG00000127838 | PNKD | paroxysmal nonkinesigenic dyskinesia | 3.477642 |
| ENSG00000119977 | TCTN3 | tectonic family member 3 | 3.467657 |
| ENSG00000112759 | SLC29A1 | solute carrier family 29, member 1 | 3.446147 |
| ENSG00000146416 | AIG1 | androgen-induced 1 | 3.399966 |
| ENSG00000183726 | TMEM50A | transmembrane protein 50A | 3.345862 |
| ENSG00000140391 | TSPAN3 | tetraspanin 3 | 3.33558 |
| ENSG00000064651 | SLC12A2 | solute carrier family 12, member 2 | 3.284861 |
| ENSG00000181704 | YIPF6 | Yip1 domain family, member 6 | 3.277872 |
| ENSG00000148175 | STOM | stomatin | 3.260948 |
| ENSG00000125107 | CNOT1 | CCR4-NOT transcription complex, subunit 1 | 3.195341 |
| ENSG00000144063 | MALL | mal, T-cell differentiation protein-like | 3.186027 |
| ENSG00000198502 | HLA-DRB5 | major histocompatibility complex, class II, DR beta 5 | 3.141387 |
| ENSG00000168216 | LMBRD1 | LMBR1 domain containing 1 | 3.13717 |
| ENSG00000105518 | TMEM205 | transmembrane protein 205 | 3.120435 |
| ENSG00000213064 | SFT2D2 | SFT2 domain containing 2 | 3.109902 |
| ENSG00000110848 | CD69 | CD69 molecule | 3.108234 |
| ENSG00000213625 | LEPROT | leptin receptor overlapping transcript | 3.098548 |
| ENSG00000101337 | TM9SF4 | transmembrane 9 superfamily protein member 4 | 3.093588 |
| **Supplementary Table 8. Cell surface transcripts highly expressed in CA125- cells** | | | |
| ENSG00000113583 | C5orf15 | chromosome 5 open reading frame 15 | 3.079131 |
| ENSG00000110651 | CD81 | CD81 molecule | 3.059954 |
| ENSG00000149932 | TMEM219 | transmembrane protein 219 | 3.05283 |
| ENSG00000134873 | CLDN10 | claudin 10 | 3.051183 |
| ENSG00000166619 | BLCAP | bladder cancer associated protein | 3.003358 |
